# Bacterial Outer Membrane Permeability Increase Underlies the Bactericidal Effect of Fatty Acids From *Hermetia illucens* (Black Soldier Fly) Larvae Fat Against Hypermucoviscous Isolates of *Klebsiella pneumoniae*

**DOI:** 10.3389/fmicb.2022.844811

**Published:** 2022-05-06

**Authors:** Heakal Mohamed, Elena Marusich, Yuriy Afanasev, Sergey Leonov

**Affiliations:** ^1^School of Biological and Medical Physics, Moscow Institute of Physics and Technology, Dolgoprudny, Russia; ^2^Institute of Cell Biophysics, Russian Academy of Sciences, Moscow, Russia

**Keywords:** *H. illucens*, free fatty acids, *Klebsiella pneumoniae*, hypermucoviscous, nosocomial isolates, colistin, NDM-1 carbapenemase-producing *Enterobacteriaceae* (CPE), MDR bacteria

## Abstract

Behind expensive treatments, *Klebsiella pneumoniae* infections account for extended hospitalization’s high mortality rates. This study aimed to evaluate the activity and mechanism of the antimicrobial action of a fatty acid-containing extract (AWME3) isolated from *Hermetia illucens* (HI) larvae fat against *K. pneumoniae* subsp. *pneumoniae* standard NDM-1 carbapenemase-producing ATCC BAA-2473 strain, along with a wild-type hypermucoviscous clinical isolate, strain *K. pneumoniae* subsp. *pneumoniae* KPi1627, and an environmental isolate, strain *K. pneumoniae* subsp. *pneumoniae* KPM9. We classified these strains as extensive multidrug-resistant (XDR) or multiple antibiotic-resistant (MDR) demonstrated by a susceptibility assay against 14 antibiotics belonging to ten classes of antibiotics. Antibacterial properties of fatty acids extracted from the HI larvae fat were evaluated using disk diffusion method, microdilution, minimum inhibitory concentration (MIC), minimum bactericidal concentration (MBC), half of the inhibitory concentration (MIC50), and bactericidal assays. In addition, the cytotoxocity of AWME3 was tested on human HEK293 cells, and AWME3 lipid profile was determined by gas chromatography-mass spectrometry (GC-MS) analysis. For the first time, we demonstrated that the inhibition zone diameter (IZD) of fatty acid-containing extract (AWME3) of the HI larvae fat tested at 20 mg/ml was 16.52 ± 0.74 and 14.23 ± 0.35 mm against colistin-resistant KPi1627 and KPM9, respectively. It was 19.72 ± 0.51 mm against the colistin-susceptible *K. pneumoniae* ATCC BAA-2473 strain. The MIC and MBC were 250 μg/ml for all the tested bacteria strains, indicating the bactericidal effect of AWME3. The MIC50 values were 155.6 ± 0.009 and 160.1 ± 0.008 μg/ml against the KPi1627 and KPM9 isolates, respectively, and 149.5 ± 0.013 μg/ml against the ATCC BAA-2473 strain in the micro-dilution assay. For the first time, we demonstrated that AWME3 dose-dependently increased bacterial cell membrane permeability as determined by the relative electric conductivity (REC) of the *K. pneumoniae* ATCC BAA-2473 suspension, and that none of the strains did not build up resistance to extended AWME3 treatment using the antibiotic resistance assay. Cytotoxicity assay showed that AWME3 is safe for human HEK293 cells at IC_50_ 266.1 μg/ml, while bactericidal for all the strains of bacteria at the same concentration. Free fatty acids (FFAs) and their derivatives were the significant substances among 33 compounds identified by the GC-MS analysis of AWME3. Cis-oleic and palmitoleic acids represent the most abundant unsaturated FAs (UFAs), while palmitic, lauric, stearic, and myristic acids were the most abundant saturated FAs (SFAs) of the AWME3 content. Bactericidal resistant-free AWM3 mechanism of action provides a rationale interpretations and the utility of HI larvae fat to develop natural biocidal resistance-free formulations that might be promising therapeutic against Gram-negative MDR bacteria causing nosocomial infections.

## Introduction

Microbial diseases have been the leading cause of mortality, and resistant microorganisms threaten public health worldwide (Vidyasagar, 2016; Khameneh et al., 2019; Biharee et al., 2020). Carbapenem-resistant Gram-negative rods are becoming a severe hazard in hospitals, leading to a serious global crisis (Makharita et al., 2020). Currently, the number of deaths has increased annually to reach 700,000 because of resistant microbes. Multidrug-resistant (MDR) microbes are expected to cause 10 million deaths per year by 2050 (Hofer, 2019). Nosocomial bacteria include *Enterococcus* spp., *Staphylococcus aureus*, *Klebsiella* spp., *Acinetobacter baumannii*, *Pseudomonas aeruginosa*, and *Enterobacter* spp., are known as the “ESKAPE” organisms (Bhagirath et al., 2019). These organisms or superbugs are returning in new forms and resistant to almost all clinically significant antimicrobials.

*Klebsiella pneumoniae*, a Gram-negative rod-shaped species of bacteria belonging to the *Enterobacteriaceae* family, is a multidrug-resistant opportunistic pathogen that causes one-third of all Gram-negative nosocomial infections such as liver abscess, septicemia, necrotizing pneumonia, urinary tract infections, surgical site infections, and dialysis-/transfusion-related infections (Joseph et al., 2021). Virulence factors of various pathogenic bacteria such as *Escherichia coli*, *S. aureus*, and *K. pneumoniae* strains include capsular polysaccharides, lipopolysaccharides, fimbriae adhesins, and siderophores, and are crucial for bacterial survival (Doorduijn et al., 2016; Algammal et al., 2020a,b). Routes for transmission of *Klebsiella* infections are very diverse, including the respiratory tract causing pneumonia, blood-eliciting bacteremia, and person-to-person contacts *via* contaminated hands. *Klebsiella* can spread very easily and rapidly but not through air. Additionally, healthcare settings that are the most vulnerable to *Klebsiella* infections for patients are on ventilators, catheters, or having surgery wounds. Infection of *K. pneumoniae* mainly occurs in the lungs, causing necrosis, inflammation, and hemorrhage in the lung tissue because of the intervention of aspirated oropharyngeal microorganisms in the lower respiratory tract. Together, hospital-acquired infections depend on the urinary tract, lower respiratory tract, biliary tract, and surgical wounds to set up colonization. The infectious dose in humans is almost not known. Besides, the incubation period is not fully understood, albeit appearing within a number of days. Thus, hospital-acquired bacterial infections caused by *K. pneumoniae* can arise in different parts of the body and in various forms of sickness depending on the process of transmission (Joseph et al., 2021; Zhu et al., 2021).

During the past decades, a new type of community-acquired hypervirulent *K. pneumoniae* (hvKP) causing life-threatening invasive infections has been described (Liu and Guo, 2019; Russo and Marr, 2019). A distinguishing feature of the majority of hvKP strains is hypermucoviscosity (HV), which is due to overproduction of capsular polysaccharides and expression of the plasmid-borne regulator of the mucoid phenotype A (*rmp*A) gene and transcriptional activator *rmp*A2 gene (Zhu et al., 2021). Among more than 80 capsular serotypes of *K. pneumoniae* identified to date, K1 and K2 serotypes were considered the cause of most severe hvKP infections (Sánchez-López et al., 2019; Li et al., 2020; Lan et al., 2021). Multidrug resistance has increased worldwide and is considered a public health threat. Several recent investigations documented the emergence of MDR pathogens from different origins, including humans, birds, cattle, and fish, increasing the need for new potent and safe antimicrobial agents. Besides, the routine application of antimicrobial susceptibility testing, which used to detect the antibiotic of choice and screening of the emerging MDR strains (Enany et al., 2018; Algammal et al., 2020c,^2021^; El-Saber Batiha et al., 2021; Kareem et al., 2021). A significant rise in the occurrence of MDR and extremely drug-resistant pathogens of the *Enterobacteaceae* group are posing a global economic threat (Hofer, 2019). Carbapenems, such as imipenem, meropenem, and ertapenem, are recommended as first-line antibiotics for treating serious infections caused by MDR bacteria (Cheng et al., 2019; Cui et al., 2019). Unfortunately, the indiscriminate and injudicious use of carbapenems has led to the appearance of bacteria resistant to these last-resort antibiotics (Aslam et al., 2018). Nowadays, the presence of NDM-1 (New Delhi Metallo-β-lactamase-1) in bacteria is one of the most clinically and epidemiologically essential resistance mechanisms to carbapenems. *K. pneumoniae* can resist multiple drugs through different mechanisms such as drug uptake limitation, drug target modulation, drug inhibition, and drug efflux pump activity (Uddin et al., 2021). Development of new drugs and newer strategies is strongly needed and emphasized by the World Health Organization (WHO) to combat antibiotic resistance (Tacconelli et al., 2018; Khameneh et al., 2019), continuously warranting the search for novel bioactive compounds in the field of natural products. More effective and more available drugs with less toxic side effects are in high demand for treatment of MDR infections (Singh et al., 2017).

Fatty acids (FAs) are molecules typically attached to sugars, glycerol, or phosphate head groups to form lipids. Lipids are necessary components of cell structures, such as cell membranes, which are main components of phospholipids, and triglycerides often compose energy stores. Fatty acids are released from fats, typically by enzymes, to become FFAs with vast and potent biological activities (Desbois and Smith, 2010). Antimicrobial activities of FFAs have roles in host defenses against potential opportunistic or pathogenic microorganisms; they can also inhibit the growth of and quickly destroy bacteria. The mechanism of the anti-virulence and ant-biofilm effects of different fatty acids from a wide range of biological sources such as algae, animals, and plants as the next generation of antibacterial agents have been reviewed elsewhere (Wille and Kydonieus, 2003; Desbois and Smith, 2010). Indeed, fatty acids are usually the active ingredients in ethnic and medical natural products (McGaw et al., 2002; Yff et al., 2002). It is necessary to repurpose FAs as an anti-biofilm agent devoid of drug resistance development to expand the existing antibiotic arsenal against biofilm-forming pathogens (Kumar et al., 2020).

FAs of insects have significant activity against pathogenic bacteria, and they are safer than chemicals or synthetic antimicrobials. Borrelli et al. (2021) reported that the lauric acid isolated from insects was considered a promising alternative for antibiotics for treating MDR bacteria. That poses the question of whether insects could serve as a potential alternative source for potent and inexpensive antimicrobials.

While *Hermetia illucens* (Black soldier fly) larvae (BSFL) are widely used to reduce and re-utilize various organic wastes to decrease animal feeding costs (Shumo et al., 2019; Pliantiangtam et al., 2021; Ramzy et al., 2021), its ability to produce FAs *via* biosynthesis pathways or by bioaccumulation from rearing diet is mainly unexplored for complete integration into the healthcare sector. Essentially, several fatty acids (i.e., decanoic, lauric, or myristic acid) were produced exclusively, while others (i.e., palmitic, palmitoleic, or oleic acid) were either partially biosynthesized or just accumulated (i.e., polyunsaturated fatty acids) by BSFL from fed (Hoc et al., 2020). Of note, even if BSFL bio-accumulated around 13% of linolenic acid from flax-enriched diets, approximately two-thirds of this fatty acid was metabolized into saturated fatty acids as lauric or myristic acid (Almeida et al., 2020). Industrial applications for *H. illucens* oil are still under development, necessitating borrowing processes from other production supply chains and requiring significant improvements to obtain good quality, activity, and cost (Almeida et al., 2020).

Characterization and modulation of the antimicrobial activity of BSFL fat concerning its FA profile are challenging and are active investigating areas of current research. *H. illucens* larvae are rich in natural active constituents with many antibacterial compounds that could potentially control the severe diseases caused by MDR bacteria. *H. illucens* larvae contain 15–49% fat, providing a rich source of lipids (Li et al., 2016). Recently, we demonstrated that FA-enriched fractions of BSFL fat possessed bactericidal activity against actual phytopathogens and MDR pathogenic fish bacteria (Marusich et al., 2020; Mohamed et al., 2021). In particular, the third acidic water-methanol extract (AWME3) was enriched in FAs and their derivatives that enabled good capacity to eradicate multidrug-resistant pathogenic fish bacteria at low doses.

In this study, we aimed to evaluate the activity and mechanism of antimicrobial action of AWME3 against hypermucoviscous strains of *K. pneumoniae* isolated in Russian hospitals in a period ranging from 2011 to 2016 including one nosocomial strain belonging to the K2 capsular types and one environmental strain belonging to the K20 capsular type. For comparison, we used the standard *K*. *pneumoniae* strain ATCC BAA-2473 with a non-HV phenotype, but producing NDM-1 carbapenemases mediating resistance has become one of the most significant current problems in drug resistance. These antibacterial activities of AWME3 against three *K. pneumoniae* strains were compared to the activity of 14 antibiotics, which belong to 10 different classes of antibiotics. Our study highlights the larval lipid part to extract new bioactive molecules effective against human MDR microbes while remaining safe to human kidney HEK293 cells. Such antibacterial agents having low toxicity and avoiding bacterial resistance are aimed to develop and explore new natural alternatives or supplements to existing antibiotics, thus warranting the development of next generation of antibacterial agents.

## Materials and Methods

### Cultures, Media, and Plastic and Chemical Reagents

Fat isolated from alive *H. illucens* larvae provided by «NordTechSad, LLC» company (Arkhangelsk, Russia) was used for this study. The HEK-293 cell line (human embryonic kidney) was obtained from the American Type Culture Collection (ATCC, Manassas, VA, United States). Cells were cultured in T75 flasks with Dulbecco’s Minimal Essential Medium (DMEM) containing 10% fetal calf serum and penicillin (100 U/ml) and streptomycin (100 μg/ml) at 37°C in 5% CO_2_ until confluence (Gibco TM; Thermo Fisher, Waltham, MA, United States). MTT (3-[4,5-dimethylthiazol-2-yl]-2,5-diphenyltetrazolium bromide) and DMSO were purchased from Sigma-Aldrich (St. Louis, MO, United States).

Reagents for extraction solution, such as hydrochloric acid (HCl) and methanol (CH_3_OH), were purchased from Thermo Fisher Scientific (Waltham, MA, United States), and Milli-Q water was obtained from Water System, Ultrapure, Millipore, and Direct-Q^®^ 3 with UV. Luria-Bertani (LB) broth, LB agar, Mullar Hinton agar (MHA), and nutrient agar (NA) were purchased from Sigma-Aldrich (St. Louis, MO, United States). Tissue culture 96-well plates (TPP; Trasadingen, Switzerland), Petri dishes (90 mm) (Pertin, Saint Petersburg, Russia), paper disks with a size of 6 mm diameter (Himedia, Mumbai, India), sterile swab (Nigbo Greetmed Medical Instruments Co., Ltd., Nigbo, China) were used for this study.

Antimicrobial susceptibility disks (6 mm) with penicillin-streptomycin (10 μg/disk), chloramphenicol (Ch) (30/disk), gentamycin (G) (10 μg/disk), and doxycycline (Dox) (30 μg/disk) were purchased from, Thermo Fisher Scientific (Waltham, MA, United States). Discs with penicillin (P) (2 U/disk), vancomycin (VA) (5 μg/disk), erythromycin (E) (60 μg/disk), rifampicin (RD) (15 μg/disk), kanamycin (K) (1,000 μg/disk), colistin (CT) (10 μg/disk), ciprofloxacin (Cip) (5 μg/disk), levofloxacin (Lev) (5 μg/disk), cefepime (Cef) (30 μg/disk), and ampicillin (Amp) (200 μg/disk) purchased from Oxoid (Basingstoke, Hampshire, United Kingdom). The different antibiotics used in this study belonged to 10 classes of antibiotics, namely, aminoglycosides (G, streptomycin, and K), ansamycins (RD), cephems (Cef), glycopeptides (VA), macrolides (E), tetracyclines (Dox), fluoroquinolones (Cip and Lev), phenicols (Ch), penicillins (P and Amp), and lipopeptides (CT).

### Bacterial Strains

The standard strain *Klebsiella pneumoniae* ATCC BAA-2473 used in this study was purchased from ATCC (American Type Culture Collection, United States). Environmental isolate *K. pneumoniae* KPM9 and clinical isolate *K. pneumoniae* KPi1627 stains were obtained from the State Collection of Pathogenic Microorganisms and Cell Cultures (SCPM, Obolensk, Russia). The bacterial strains were stored in sterile glycerol stock (30%, v/v) at –80°C. A single colony from each strain was inoculated in 10 ml of the LB broth, incubated overnight at 37°C by shaking at 210 rpm/min. The overnight culture was adjusted to half of the McFarland standard (1 × 10^8^ CFU/ml) and used in susceptibility assays. All the experiments were implemented in a sterile cabinet (Safe Fast Elite, Ferrara, Italy).

### *Hermetia illucens* Larvae Fat Extraction

Fat isolated from alive *H. illucens* larvae with age of 15 days old was provided by the NordTechSad, LLC company (Arkhangelsk, Russia) and used for this study. The *H. illucens* larvae fat was extracted according to our previous study (Mohamed et al., 2021). Briefly, 3 g of larvae fat was subjected to three rounds of extraction using an extraction solution composed of water (Milli Q quality), methanol (99.9%, HPLC grade), and hydrochloric acid (37%) with a ratio of 90:9:1, v/v/v at very low acidic pH (<1, 0). Since our previous study demonstrated that AWME3 was the most potent among other extracts against pathogenic *Aeromonas* **sp.** (Mohamed et al., 2021), the third extract AWME3 was selected for our experiments in this study.

### Antimicrobial Susceptibility Testing

Antimicrobial susceptibility was determined using the disk diffusion technique according to the National Committee for Clinical Laboratory Standards 2018 guidelines (Clinical and Laboratory standards Institute [CLSI], 2018; Algammal et al., 2020d). All strains were obtained from a single colony, and the overnight cultures were adjusted to 0.5 McFarland standard (1 × 10^8^ CFU/ml) and tested against a panel of 14 antibiotics belonging to 10 different classes using antibiotic disks in Mueller-Hinton (MH) agar. The antimicrobial agents included penicillin-streptomycin (200 U/ml-200 μg/ml), gentamycin (200 μg/ml), doxycycline (600 μg/ml), chloramphenicol (600 μg/ml), ciprofloxacin (100 μg/ml), levofloxacin (100 μg/ml), cefepime (600 μg/ml), and ampicillin (4,000 μg/ml), where 50 μl of each antibiotic was loaded on 6 mm disks, and then the disks were dried under sterilized cabinet conditions for 45 min and transferred to agar plates. Disks loaded with penicillin (2 U/disk, P), vancomycin (5 μg/disk, VA), erythromycin (60 μg/disk, E), rifampicin (15 μg/disk, RD), kanamycin (1,000 μg/disk, K), and colistin (10 μg/disk, CT) were picked up and transferred to bacterial agar plates and pressed gently. Additionally, the minimum inhibitory concentration for these antibiotics was determined according to a microdillution assay; therefore, the minimum bactericidal concentration was determined based on the MIC assay. The strains of *K. pneumoniae* were classified as sensitive (S), intermediate (I), and resistant (R) according to the Clinical and Laboratory standards Institute [CLSI] (2018) breakpoints for *Enterobacteria*. Inhibition zone diameters (IZD) were measured and compared to measures of the CLSI guidelines (Clinical and Laboratory standards Institute [CLSI], 2018). Strains resistant to at least one agent in three or more antimicrobial categories were defined as multidrug-resistant (MDR), strains which are resistant to all categories except one or two were defined as extensively drug resistant (XDR), while strains show resistance to all categories of antibiotics was defined as pandrug resistant (PDR) (Magiorakos et al., 2012). All data values were recorded at 12 and 24 h of incubation.

### Minimum Inhibitory Concentration

Minimum inhibitory concentration (MIC) was assessed using a 2-fold broth micro-dilution assay according to the guidelines of the Clinical Laboratory Standards Institute (Clinical and Laboratory standards Institute [CLSI], 2015; Popović et al., 2020). Experiments were carried out on 96-well microtiter plates using a series of 2-fold dilutions of the AWME3 extract as follows: 8 mg/ml AWME3 was tested in a range from 2,000 to 15.63 μg/ml. Likewise, the positive control (doxycycline) that was susceptible to the all the strains was serially 2-fold diluted in the range of 50–0.195 μg/ml. Microplates were sealed and incubated at 37°C for 24 h with shaking at 200 rpm/min. The value of MIC was recorded as the lowest concentration of the AWME3 extract, showing no detectable bacterial growth in the wells. All the experiments were performed in triplicate.

### Minimal Bactericidal Concentration

Minimal bactericidal concentration (MBC) value was defined as the minimum concentration of the AWME3 extract, which kills 99.9% of the starting inoculum. For MBC determination, an aliquot of 40 μl of bacteria from the wells corresponding to 1x MIC, 2x MIC, and 4x MIC were plated and then incubated for 48 h at 37°C (Popović et al., 2020). MBC was defined as the lowest concentration of the tested AWME3 of BSFL fat that showed no growth on the surface of the Petri dishes with agar. The MBC of AWME3 was compared with the MBC of the positive control (DOX) used against the *K. pneumoniae* ATCC BAA-2473, *K. pneumoniae* KM9, and *K. pneumoniae* KPi1627 bacteria. All the tests were performed in triplicate. Every experiment was repeated at three different times.

### Half of the Minimum Inhibitory Concentration

Half of the minimum inhibitory concentration (MIC50) was defined as the minimum concentration of AWME3, which inhibits 50% of total bacterial growth. A total of 100 μL of the AWME3 extract was serially diluted in the MH broth and ranged from 2,000 to 15.63 μg/ml in a 96–well microtiter plate and had a final volume of 200 μl (Balkrishna et al., 2019). The reference control doxycycline was tested against clinical strains and was serially diluted in the range of 50–0.195 μg/ml. MIC_50_ was determined at 12 and 24 h by measuring the OD_600_ for tested strains using a CLARIO star microplate reader (BMG LABTECH, Ortenberg, Germany). All the experiments were performed in triplicates. MIC_50_ was determined using the GraphPad Prism 7 software using the non-linear regression mode.

### Benchmarking of the Bactericidal and Bacteriostatic Activities of Acidic Water Methanol Extract 3

Minimum Inhibitory Concentration (bacteriostatic) and MBC (bactericidal) methods were used to evaluate the antimicrobial effect of AWME3 on laboratory *K. pneumoniae* ATCC BAA-2473 and clinical *K. pneumoniae* KPM9, and *K. pneumoniae* KPi1627 strains. If the ratio of MBC:MIC values was equal to 1 or 2, the antimicrobial effect was considered as bactericidal. However, if the ratio of MBC:MIC value was equal to 4 or above, AWME3 activity was considered as bacteriostatic. The activity of the reference control (DOX) was evaluated with the same token (Konaté et al., 2012).

### Evaluation of Antibiotic Resistance to the Acidic Water Methanol Extract 3 Extract

Resistance to AWME3 isolated from *H. illucens* larvae fat was determined according to Duan et al. (2021), with minor changes (Duan et al., 2021). The tested microorganisms *K. pneumoniae* ATCC BAA-2473, *K. pneumoniae* KPM9, and *K. pneumoniae* KPi1627 were subcultured with a sub-MIC (125 μg/ml) concentration of AWME3 and the positive control (P/S) for 16 consecutive days to investigate their ability to develop resistance to the tested drugs. Briefly, bacterial cultures were harvested in the logarithmic phase, washed three times with PBS (pH 7.4), and diluted to 5 × 10^5^ (CFU/ml) in a fresh LB medium containing 0.5x, 1x, 2x, and 4x MIC concentrations of AWME3 or an antibiotic, and incubated at 37°C for 24 h. Aliquots of cultures from the second highest concentration (0.25x MIC) of AWME3 or an antibiotic that allowed growth (OD_600_ > 0.1) were diluted 1:100 in fresh an LB medium containing the same set of MIC concentrations based on the most recently observed MIC. With increasing concentrations of AWME3 or the antibiotic, serial passaging was repeated for 16 days for every strain.

### Assessment of Acidic Water Methanol Extract 3 Impact on Bacterial Membrane Permeability

Relative electric conductivity (REC) value was calculated according to Kong et al. (2008), with minor changes, and used as a measure of bacterial membrane permeability. *K. pneumoniae* ATCC BAA-2473 was cultured in 10 ml of the LB broth at 37°C for 10 h under shaking conditions, and then centrifuged at 4,200 rpm/min for 10 min. Then, the pellet of the bacteria was washed with 5% of glucose in water until its REC value was equal or near the REC value of the washing buffer (5% glucose), generating a so-called isotonic bacterial solution. The AWME3 at four different concentrations (0.5x MIC, 1x MIC, 2x MIC, and 4x MIC) were diluted in 5% glucose, and electric conductivities of the mixtures were measured using a conductometer (Ionomer, Moscow, Russia) and marked as *L_1_.* Then, the same concentrations of AWME3 were added into the isotonic suspension of *K. pneumoniae* ATCC BAA-2473 at 10^8^ CFU/ml. After intensive mixing, the samples were incubated at 37°C for 8 h, and the conductivities were measured and marked as *L*_2_. The bacteria 5% glucose were boiled in the water bath for 5 min and served as the positive control, using their conductivity marked as *L*_0_. Relative electric conductivity was calculated according to the formula (%) = (*L*_2_ -*L*_1_)/*L*_0_ × 100, reflecting bacterial membrane permeability value.

### 3-(4,5-Dimethylthiazol-2-yl)-2,5-Diphenyltetrazolium Bromide Cytotoxicity Assay

3-(4,5-dimethylthiazol-2-yl)-2,5-diphenyltetrazolium bromide (MTT) assays were carried out on a 96-well plate based on the method described by Somaida et al. (2020). Briefly, 100 μL of AWME3 was serially diluted in 2 folds of dilution in the DMEM medium to get the final concentrations (1,000, 500, 250, 125, 62.5, 31.25, 15.625, and 7.813 μg/ml), and then added to a 96-well plate, which contained 100 μl of HEK293 cells, and the plate was incubated for 24 h at 37°C and 5% CO_2_. After that, 20 μl of 5 mg/ml MTT was added to each well (1 × 10^4^ cells/well). MTT specific activity was determined after 2 h of incubation at 37°C and 5% CO_2_; then, 100 μl of DMSO was added into each well of the 96-well plate. The plate was shaken for 10 min to dissolve formazan crystals. Data were presented as a percentage of viability, when compared with untreated cells. Furthermore, the blank included during MTT incubation included wells without cells (DMSO + MTT). Viability was calculated based on comparison with the absorbance of untreated and treated cells. IC_50_ values were obtained from the concentration of the AWME3 of *H. illucens* larvae fat that induced 50% inhibition of cell growth using the Graph Pad prism 7 software. The optical density (OD) in each well was determined with a microplate reader, Clariostar, using an absorption spectrum of 580 nm.

### Gas Chromatography-Mass Spectrometry Analysis

Gas Chromatography-Mass Spectrometry (GC-MS) analyses of AWME3 were carried out using a GC-MS-QP2010 ultra mass spectrometer (Shimadzu, Canby, OR, United States) equipped with a fused silica column, capillary column DB-5 ms measuring 30 mm × 0.25 mm with a thickness of 0.25 mm coated with a non-polar silphenylene polymer with polarity of 5% diphenyl and 95% dimethylpolysiloxane in stationary phase (Restek, Bellefonte, PA, United States). Pure helium gas (99.99%) was used as the carrier gas with a linear flow rate ranging from 1 to 15 ml/min, and column head pressure was 50.4 kPa. For GC–MS spectral detection, an electron ionization energy method was adopted with high ionization energy of 70 eV (electron Volts), with 0.2 s of scan time and fragments ranging from 40 to 600 m/z. Analysis procedure was as follows: 1 μl of AWME3 extract was injected with an autosampler injector automatically. Injector and detector temperatures were maintained at 280 and 250°C, respectively. Temperature program was initially set at 40°C, held for 1 min, and then increased to 210°C at a rate of 15°C/min, held for 0 min, and then it was increased to 216°C at a rate 5°C/min and held for 0 min. Then, the temperature was increased to 300°C at a flow rate of 40°C/min and held for 14.87 min. The contents of AWME3 present in the sample were identified based on comparison of their retention time (min), peak area, peak height, and mass spectral patterns with those in spectral databases of authentic compounds stored in the National Institute of Standards and Technology (NIST-08) library. Compounds with chromatogram peaks matched with similarity index (SI) ≥ 70% in the NIST-8 library were ascertained.

### Statistical Analysis

Statistical analysis performed by two-way analysis of variance of repeated measurements (Two-way RM-ANOVA) to determine the statistically significant differences between treatments of the AWME3 susceptibility against tested bacteria strains compared to positive control. Differences between means were compared by Dunnett’s *post-hoc* test. Results of fatty acid profiles were analyzed by ordinary one-way ANOVA, and Tukey’s *post-hoc* test was conducted to determine significant differences between treatment means. All the data were assessed by the standard deviation (SD) using software GraphPad Prism 7 (GraphPad Software Inc., San Diego, CA, United States). Statistical significance level was *P* < 0.05.

## Results

### Multidrug-Resistant Assessment of Human *Klebsiella pneumoniae* Strains

The antibiotic resistance phenomenon represents one of the most critical public health issues, and antibiotic-resistant genes are considered the emerging pollutant of the environment (Sabri et al., 2020). The disk diffusion method was used to determine the antimicrobial AWME3 susceptibility effect on three bacterial strains: *K. pneumoniae* ATCC BAA-2473, *K. pneumoniae* KPM9, and *K. pneumoniae* KPi1627. IZD values were interpreted by comparison to *E. coli* values, because there are no guideline breakpoints for *K. pneumoniae* in the CLSI guidelines. The breakpoints of *E. coli* are recommended and used for other *Enterobacteriaceae* such as the *Klebsiella* species (Le et al., 2021). To evaluate the susceptibility of the three tested strains of *K. pneumoniae*, we determined IZDs by disk assay, MIC. MBC values were determined by microdilluition method (Supplementary Table 1), which is recommended by Clinical and Laboratory standards Institute [CLSI] (2018), *K. pneumoniae* ATCC BAA-2473 showed resistance to 12, while *K. pneumoniae* KPi1627 and *K. pneumoniae* KPM9 were resistant to only six of 14 tested antimicrobials (Supplementary Figure 1, Table 1, and Supplementary Table 1).

**TABLE 1:** Antibiotic susceptibility of human *Klebsiella pneumoniae* strains.

Antibiotics	IZD (mm)								
	KPATCC BAA-2473		KPi 1627		KPM9		Breakpoint (mm)		
	12 h	24 h	12 h	24 h	12 h	24 h	R	I	S
G	0 (R)	0 (R)	16.55 ± 2.36 (S)	16.1 ± 0.2 (S)	16.93 ± 1.18 (S)	16.72 ± 2 (S)	≤12	13–14	≥15
Ch	10.27 ± 0.07 (R)	9.92 ± 1 (R)	21.55 ± 0.19 (S)	21.7 ± 1.9 (S)	18.33 ± 2.1 (S)	18.15 ± 1.6 (S)	≤12	13–17	≥18
K	11.1 ± 0.72 (R)	10.87 ± 0.27 (R)	21.33 + 0.47 (S)	22.36 + 1.84 (S)	22.36 + 1.84 (S)	20.7 + 1.6 (S)	≤13	14–17	≥18
DOX	18.48 ± 2.3 (S)	18.1 ± 1.2 (S)	19.18 ± 0.8 (S)	18.62 ± 0.35(S)	18.68 ± 0.9 (S)	17.43 ± 0.8 (S)	<10	11–13	≥14
P/S	0 (R)	0 (R)	14.7 ± 0.42 (I)	14.45 ± 0.44 (I)	15.56 ± 0.37 (S)	14.88 ± 0.23 (I)	≤11	12–14	≥15
CT	11.6 ± 0.14 (S)	11.35 ± 0.49 (S)	8.52 ± 0.38 (R)	8.12 ± 0.26 (R)	9.74 ± 0.76 (R)	9.23 ± 0.26 (R)	≤10	-	≥11
RD	0 (R)	0 (R)	9.38 ± 0.47 (R)	9 ± 0.79 (R)	10.36 ± 0.86 (R)	10.5 ± 0.75 (R)	≤16	17–19	≥20
E	9.5 ± 1.13 (R)	10.1 ± 1.5 (R)	9.97 ± 0.71 (R)	9.9 ± 0.75 (R)	10.57 ± 0.53 (R)	10.35 ± 0.41 (R)	≤13	14–22	≥23
VA	0 (R)	0 (R)	0 (R)	0 (R)	0 (R)	0 (R)	-	-	≥15
P	0 (R)	0 (R)	0 (R)	0 (R)	0 (R)	0 (R)	≤28	-	≥29
Amp	0 (R)	0 (R)	12.08 ± 0.7 (R)	11.62 ± 0.44 (R)	12.48 ± 0.44 (R)	11.78 ± 0.6 (R)	≤13	14–16	≥17
Cef	0 (R)	0 (R)	29.35 ± 0.64 (S)	28.9 ± 0.82 (S)	30.9 ± 0.61 (S)	30.48 ± 0.56 (S)	≤18	-	≥25
Cip	0 (R)	0 (R)	29.23 ± 0.85 (S)	28.85 ± 0.39 (S)	30.53 ± 0.33 (S)	29.95 ± 0.24 (S)	≤15	16–20	≥21
Lev	0 (R)	0 (R)	29.1 ± 0.57(S)	28.87 ± 0.66(S)	29.33 ± 0.4(S)	29 ± 0.33(S)	≤13	14–16	≥17

*G, gentamicin; Ch, chloramphenicol; K, kanamycin; DOX, doxycycline; P/S, penicillin-streptomycin; CT, colistin; RD, rifampicin; E, erythromycin; VA, vancomycin; P, penicillin; Amp, ampicillin; Lev, levofloxacin; Cip, ciprofloxacin; Cef, cefepime; R, resistant; I, intermediate; S, susceptible; (0) means no inhibition zone around the disc on the plate; (-), not determined.*

All the *K. pneumoniae* strains demonstrated sustainable 100% resistance to penicillin, vancomycin, erythromycin, and rifampicin strains during 12 h and 24 h of incubations. All the tested *K. pneumoniae* strains isolated from different sources exhibited high susceptibility to doxycycline (Supplementary Figure 1 and Supplementary Table 1), being the rationale of using it as a positive control in this study. *K. pneumoniae* ATCC BAA-2473 was 100% resistant to gentamicin and penicillin, where no IZD occurred around the discs at 12 and 24-h of the incubations and possessed high resistance to kanamycin and chloramphenicol confirmed by the IZD in the disk diffusion assay (Supplementary Figure 1). Moreover, the MIC determined *via* the microdillution assay shows that *K. pneumoniae* KPi1627 and *K. pneumoniae* KPM9 were resistant to six antimicrobial drugs belonging to five classes, and that *K. pneumoniae* ATCC BAA-2473 exhibited highest resistance compared to the other two strains, where it was resistant to all the tested antibiotics except for two categories (colistin and doxycycline) (see Supplementary Table 1). Of note, both the wild-type nosocomial KPi1627 strain and the environmentally isolated KPM9 strain were resistant to colistin, the antibiotic of last resort that is broadly active against Gram-negative bacteria despite having side effects such as nephrotoxicity and ototoxicity. In contrast, the *K. pneumoniae* ATCC BAA-2473 strain was susceptible to this antibiotic but show high resistance to the fluoroquinolone (ciprofloxacin and levofloxacin), and cephem (cefepime) groups. On the other hand, *K. pneumoniae* KPi1627 and *K. pneumoniae* KPM9 showed significant susceptibility to the same categories of antibiotics (Cip, Levo, and Cef) (Supplementary Table 1). Based on the ratio of MBC/MIC, all the antibiotic categories were classified as bactericidal or bacteriostatic. Thus, if the ratio MBC/MIC ≥ 4, bacteria were bacteriostatic, while if MBC/MIC ≤ 2, bacteria were bactericidal. In our study, chloramphenicol, doxycycline, rifampicin, ampicillin, and cefepime showed bacteriostatic activity against *K. pneumoniae* KPi1627. The antibacterial agents chloramphenicol and doxycycline displayed bacteriostatic properties against the *K. pneumoniae* KPM9 strain, while five important antibiotics, namely, chloramphenicol, doxycycline, colistin, levofloxacin, and cefepime were bacteriostatic against *K. pneumoniae* ATCC BAA-2473 (Supplementary Table 1). Based on our findings, colistin and doxycycline were recommended for treatment of *K. pneumoniae* ATCC BAA-2473, while gentamycin, kanamycin, penicillin-streptomycin, ciprofloxacin, levofloxacin, and cefepime were recommended for treatment of both the *K. pneumoniae* KPi1627 and *K. pneumoniae* KPM9 strains. The percentage of antibiotic resistance of *K. pneumoniae* ATCC BAA-2473, *K. pneumoniae* KPi1627, and *K. pneumoniae* KPM9 to the 14 different antibiotics were 83.33, 42.86, and 42.86%, respectively. Hence, the *K. pneumoniae* KPi1627 and *K. pneumoniae* KPM9 strains were classified as multidrug-resistant (MDR) because they were resistant to two or more antibiotics. *K. pneumoniae* ATCC BAA-2473 was classified to be extensive drug resistant (XDR), because it was resistant to all the antibiotics except for two categories (doxycycline and colistin) of the total 14 used in our study (Supplementary Table 2).

### Acidic Water Methanol Extract 3 Obtained by Sequential Extraction From *Hermetia illucens* Larvae Fat

**FIGURE 1:**
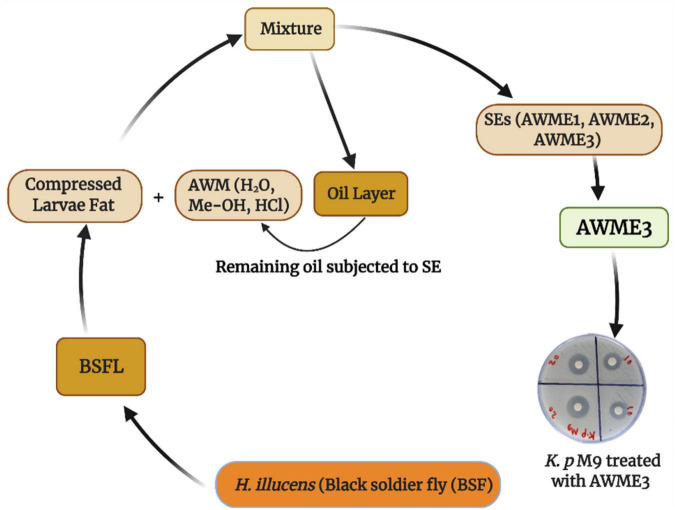
Schematic diagram for isolation of bioactive compounds from *H. illucens* larvae fat using sequential extraction procedure.

Recently, we demonstrated the advantage of using consecutive extracts of the HI larvae fat where bioactive compounds from the AWME3 extract showed higher level of antimicrobial activity against pathogenic fish bacteria than the first two extracts (Mohamed et al., 2021). The schematic diagram of the workflow shows the developed methodology for obtaining AWME3 using sequential extraction approach from the same biomass of the HI larvae fat (Figure 1).

### Antibacterial Activity of Acidic Water Methanol Extract 3 Against Human Pathogenic Bacterial Strains

Different concentrations of AWME3 were tested against the *K. pneumoniae* strains by disk diffusion assay to assess antibacterial activity. All the tested Gram-negative bacterial strains were treated with 1.25, 2.5, 5, 10, and 20 mg/ml concentrations of AWME3, which were loaded as 50-μl aliquots in 6-mm-diameter discs. IZD was determined by measuring the inhibition halos induced by AWME3 around the discs at 12 and 24 h of incubation time (Table 2 and Supplementary Figure 2). For the first time, AWME3 extract activity was evaluated against clinical and environmental isolates of *K. pneumoniae*, such as *K. pneumoniae* KPi1627 and *K. pneumoniae* KPM9 strains, and compared with the standard *K. pneumoniae* ATCC BAA-2473 strain.

**TABLE 2:** Inhibition zone diameters (IZDs) caused by the AWME3 of *H. illucens* larvae fat against *K. pneumoniae* strains.

AWME3 (mg/mL)	IZD (mm)					
	KP ATCC BAA-2473		KPM9		KPi1627	
	12 h	24 h	12 h	24 h	12 h	24 h
20	22.8 ± 0.47	19.72 ± 0.51	18.7 ± 0.52	16.52 ± 0.74	14.83 ± 0.49	14.23 ± 0.35
10	14.7 ± 0.26	13.88 ± 0.35	13.6 ± 0.56	12.98 ± 0.39	11.6 ± 0.34	10.5 ± 0.37
5	12.73 ± 0.21	10.78 ± 0.33	10.25 ± 0.4	9.48 ± 0.28	9.43 ± 0.24	8.67 ± 0.24
2.5	11.17 ± 0.24	9.25 ± 0.54	8.1 ± 0.33	7.67 ± 0.25	7.45 ± 0.32	7.18 ± 0.19
1.25	8.9 ± 0.45	8.06 ± 0.52	7.15 ± 0.27	6.63 ± 0.2	6.86 ± 0.19	6.5 ± 0.23
P.C (DOX)	18.48 ± 2.3	18.1 ± 1.2	19.18 ± 0.8	18.62 ± 0.35	18.68 ± 0.9	17.43 ± 0.8
N.C	ND	ND	ND	ND	ND	ND

*ND, not determined; N.C, negative control; P.C (DOX), positive control (doxycycline) at a concentration of 600 μg/ml (30 μg/disk).*

**FIGURE 2:**
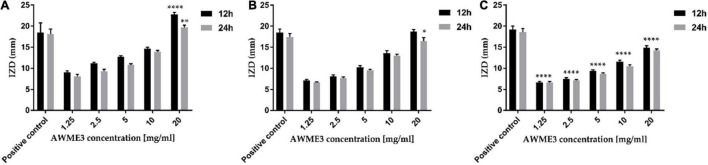
Antimicrobial sensitivity of AWME3 against **(A)** *K. pneumoniae* ATCC BAA-2473, **(B)** *K. pneumoniae* KPM9, and **(C)** *K. pneumoniae* KPi1627 strains. The bacteria strains were subjected to concentrations of 1.25, 2.5, 5, 10, and 20 mg/mL of AWME3 from BSFL fat. The IZD values were measured after 12 and 24 h of incubation at 37°C. Doxycycline (DOX) used as a positive antibacterial control. All values are represented as mean ± SD, in triplicate (*n* = 3). Data were analyzed by two-way ANOVA, followed by Dunnett’s Test. Data represented as significant difference as compared to positive control and *p*-value was ranged between. *p = 0.0138, **p = 0.0062, ****p < 0.0001.

The ANOVA results indicated a significant difference among the mean inhibition halos obtained by treating the different human pathogenic bacterial strains with AWME3 when compared to the IZD caused by the reference antibiotic (Dox) (*p* ≤ 0.05). The most significant inhibition zone size belonged to *K. pneumoniae* ATCC BAA-2473 and *K. pneumoniae* KPM9, indicating their highest susceptibility to the AWME3 obtained from the natural fat of *H. illucens* larvae. Their IZD value was 19.72 ± 0.51 and 16.52 ± 0.74 mm, respectively, when treated with 20 mg/ml at 24 h of incubation; compared to the positive control (Dox) it was 18.1 ± 1.2 and 18.62 ± 0.35 mm, respectively. The *K. pneumoniae* KPi1627 strain was the least susceptible and recorded a significant difference (^****^p < 0.0001) compared to the reference control at 12 and 24 h of incubation time (Figure 2). Our results show that all these MDR strains isolated from different sources can be inhibited in a dose-dependent manner by AWME3. Moreover, all the tested *K. pneumoniae* strains were eradicated and killed at a 20 mg/ml concentration of AWME3.

### Benchmarking Antibacterial Activity of Acidic Water Methanol Extract 3 Against Human *Klebsiella pneumoniae* Strains

Table 3 shows the MICs of AWME3 for all the tested bacterial strains obtained after 24 h of incubation. AWME3 inhibited *K. pneumoniae* ATCC BAA-2473, *K. pneumoniae* KPM9, and *K. pneumoniae* KPi1627 growth at a MIC concentration 250 μg/ml. Doxycycline (positive control) inhibited the growth of *K. pneumoniae* ATCC BAA-2473 bacteria at MIC 6.25 μg/ml, while inhibition of *K. pneumoniae* KPM9 and *K. pneumoniae* KPi1627 growth was obtained at MICs 3.12 and 1.56 μg/mL, respectively. All the tested strains were treated with different concentrations of the positive control in the range 0.195–50 μg/ml. MBC is defined as the lowest concentration, killing 99.99% of the tested bacteria strains after 48 h of incubation time. AWME3 demonstrated equal MBC 250 μg/ml for all the tested human *K. pneumoniae* pathogens. In contrast, MBC 50 μg/ml for doxycyclin was the highest for the *K. pneumoniae* ATCC BAA-2473 strain compared to the MBC 12.5 μg/ml for *K. pneumoniae* KPM9 and MBC 6.25 μg/mL for the *K. pneumoniae* KPi1627 isolates. The ratio of MBC/MIC did not exceed 2 for AWME3 while ranging between 4 and 8 for the positive control (DOX). These results demonstrated that AWME3 has sustainable bactericidal activity comparable to the reference antibiotic control (Dox) that exhibited bacteriostatic activity against all the tested *K. pneumoniae* strains. Furthermore, the MBC (250 μg/ml) of AWME3 was lower than the MBC of nine antibiotics (gentamycin, penicillin-streptomycin, kanamycin, rifampicin, erythromycin, penicillin, ampicillin, levofloxacin, and ciprofloxacin) used for testing against *K. pneumoniae* ATCC BAA-2473, where their MBC ranged between 312.5 and ≥1,250 μg/ml (Supplementary Table 1).

**TABLE 3:** Antibacterial activity measured by the MIC and MBC of AWME3 against *K. pneumoniae* strains.

Antibacterial agent	Activity	Concentration (μg/ml)		
		KP ATCC BAA-2473	KPM9	KPi 1627
AWME3	MIC	250	250	250
	MBC	250	250	250
	MBC/MIC (ratio)	1.0	1.0	1.0
Positive control (DOX)	MIC	6.25	3.12	1.56
	MBC	50	12.5	6.25
	MBC/MIC (ratio)	8.0	4.0	4.0

### Determination of the Half of the Minimum Inhibitory Concentration Values of Acidic Water Methanol Extract 3

**FIGURE 3:**
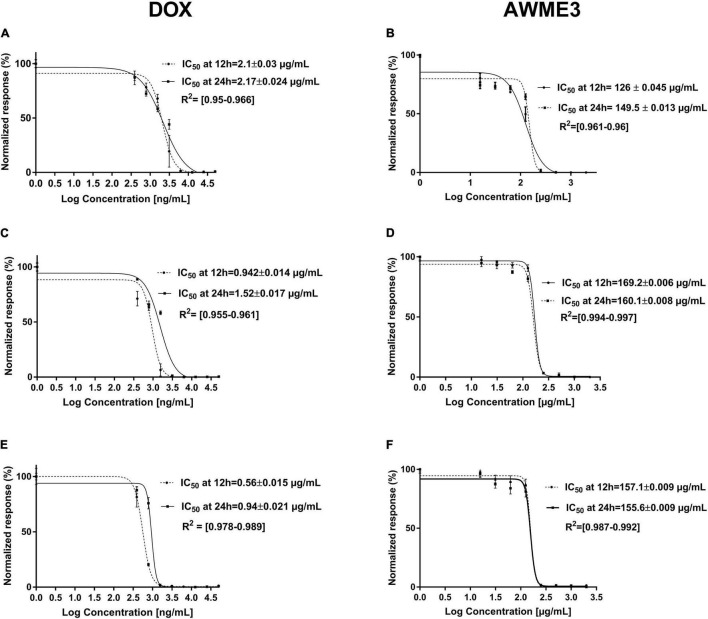
The MIC_50_ (IC_50_ value in the figure legends) of *K. pneumoniae* strains treated with AWME3 from the larvae fat compared to the doxycycline (DOX) as a positive control. The MIC_50_ values were calculated based on the turbidimetric assay data and compared to the positive control (DOX). The planktonic bacteria turbidity was assessed for **(A,B)** *K. pneumoniae* ATCC BAA-2473; **(C,D)** *K. pneumoniae* KPM9; **(E,F)** *K. pneumoniae* KPi1627 strains at 12 and 24 h incubation with **(A,C,E)** DOX; **(B,D,F)** AWME3. The MIC_50_ values were calculated using the non-linear regression mode of Graph pad Prism 7 (Graph Pad Software Inc., San Diego, CA, United States). The (IC_50_)MIC_50_ values are the average of three independent experiments ± standard deviation error mean (SEM).

Half of the minimal inhibitory concentration (MIC50) values of the AWME3 and the positive control against human *K. pneumoniae* strains are shown in Table 4 and Figure 3.

**TABLE 4:** Half of the inhibitory concentration (MIC_50_) values of AWME3 and positive control against MDR *K. pneumoniae* strains.

Bacteria strains	MIC50 (μg/mL)			
	Positive control		AWME3	
	12 h	24 h	12 h	24 h
*K. pneumoniae* ATCC BAA-2473	2.1 ± 0.03	2.17 ± 0.024	126 ± 0.045	149.5 ± 0.013
*K. pneumoniae* KPM9	0.942 ± 0.014	1.52 ± 0.017	169.2 ± 0.006	160.1 ± 0.008
*K. pneumoniae* KPi1627	0.56 ± 0.015	0.94 ± 0.021	157.1 ± 0.009	155.6 ± 0.009

Acidic Water Methanol Extract 3 showed MIC50 values ranging from 149.5 ± 0.013 to 160.1 ± 0.008 μg/ml after 24 h of incubation against all the tested bacterial strains. Of note, the MIC50 values of AWME3 decreased from 12 (169.2 ± 0.006–157.1 ± 0.009 μg/ml) to 24 h (160.1 ± 0.008–155.6 ± 0.009 μg/ml) the against *K. pneumoniae* KPM9 and *K. pneumoniae* KPi1627 strains, respectively. The MIC50 values of doxycycline increased by 60% from 12 to 24 h against clinical KPi1627 and environmental KPM9 isolates, while the reference strain KP ATCC BAA-2473 demonstrated only a 10% MIC_50_ increase (Table 4 and Figure 3). *K. pneumoniae* ATCC BAA-2473 was the most resistant to different concentrations of the positive control (Dox) compared to both wild-type *K. pneumoniae* isolates (Table 4). AWME3 possessed significant concentration-dependent inhibition, indicating high efficacy against MDR *K. pneumoniae* strains.

### Assessment of Human Bacterial Pathogen Resistance to Acidic Water Methanol Extract 3 Treatment

**FIGURE 4:**
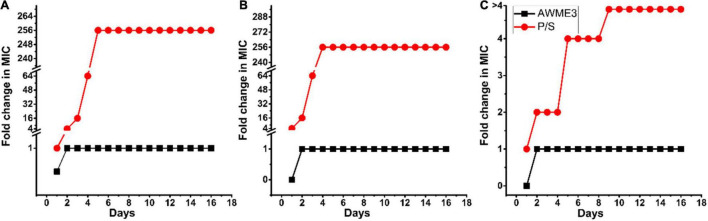
*Klebsiella pneumoni*ae strains resistance assessment. Resistance acquisition monitored during 16 serial passages (16 days) in the presence of sub-MIC (0.5 × MIC) of AWME3, and positive control (P/S) for **(A)** *K. pneumoniae* KPi1627, **(B)** *K. pneumoniae* KPM9, and **(C)** *K. pneumoniae* ATCC BAA-2473. The Y-axis represents the highest bacterial concentration during cell passaging. The figures are representative of three independent experiments.

Antibacterial resistance was determined by sub-culturing the tested MDR bacteria strains with the sub-MIC (125 μg/ml) concentration of the AWME3 extract or the antibiotics for 16 consecutive days. The development of antimicrobial resistance is defined as more significant than a 4-fold increase in its initial MIC (Duan et al., 2021). Our results demonstrated that the MIC values of AWME3 remained unchanged for the tested bacterial strains, namely, *K. pneumoniae* ATCC BAA-2473, *K. pneumoniae* KPM9, and *K. pneumoniae* KPi1627, indicating that the human pathogens did not acquire resistance to active compounds in the AWME3 extract (Figure 4). In contrast, serial passaging of all the tested bacterial strains treated with the reference antibiotic (P/S) showed more than 4-fold higher than the initial MICs (Figure 4), demonstrating a significant increase in antibiotic resistance. Indeed, the P/S initial MIC (9.77 μg/ml) raised to 256x MIC (2,500 μg/ml) the for *K. pneumoniae* KPi1627, and *K. pneumoniae* KPM9 strains. For the *K. pneumoniae* ATCC BAA-2473 strain, resistance increased to 4× MIC (>5,000 μg/ml) from the initial MIC (1,250 μg/mL). Thus, *K.pneumoniae* ATCC BAA-2473, *K. pneumoniae* KPM9, and *K. pneumoniae* KPi1627 did not show resistance in response to AWME3 extended treatment.

### Bacterial Cell Membrane Permeability

**FIGURE 5:**
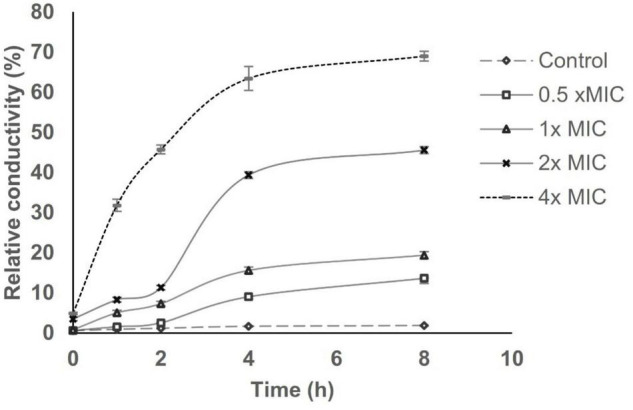
Effect of AWME3 concentrations on the cell membrane perme ability of *K. pneumoniae* ATCC BAA-2473 strain. Planktonic bacteria sus pension at 10^8^ CFU/mL was subjected to various concentrations of AW ME3 ranged from 0.5 (125 μg/mL) to 4x MIC (1,000 μg/mL), and incubated for 8 h at 37°C. REC was calculated at 0, 1, 2, 4, and 8 h based on the values of electrical conductivity. Bacteria without AWME3 treatment was considered as negative control. All values presented as the mean of three independent experiments ± SD.

Cell membrane permeability was determined based on relative electric conductivity (REC). Figure 5 shows the effect of AWME3 from the *H. illucens* larvae fat on the REC of the *K. pneumoniae* ATCC BAA-2473 strain. The REC of the control (non-treated) planktonic bacteria recorded a marginal increase, which might be due to the standard lysis and death of the bacteria. AWME3 treatment in the concentration range of 0.5–2x MIC demonstrated a biphasic effect on REC with a smooth lift by the second hour followed by a more pronounced increase by the fourth hour with almost flattened change within the next 4 h. Indeed, AWME3-induced REC gradually increased within the first 4 h, reaching a 1.5-fold rise to 13.62 ± 1.29 and 19.38 ± 0.87% against 0.5 and 1x MIC, respectively, in 8 h. A more pronounced REC increase of up to 45.57 ± 0.87 and 69 ± 1.21% was caused by the 2x MIC and the 4x MIC, respectively, with the same incubation time. Notably, the 4x MIC induced a rapid sharp increase by the first hour of incubation, recording an REC of 31.82 ± 1.21%. By the eighth hour of AWME3 exposure, the REC of *K. pneumoniae* ATCC BAA-2473 at the control 0.5, 1, 2, and 4× MIC was 1.86 ± 0.12, 13.62 ± 1.29, 19.38 ± 0.87, and 69 ± 1.21%, respectively. Hence, the AWME3 dose-dependently increased the REC of the treated bacteria suggesting leakage of bacterial electrolytes due to disruption of cell permeability caused by AWME3 treatment with the incubation time.

### Cytotoxicity of Acidic Water Methanol Extract 3

**FIGURE 6:**
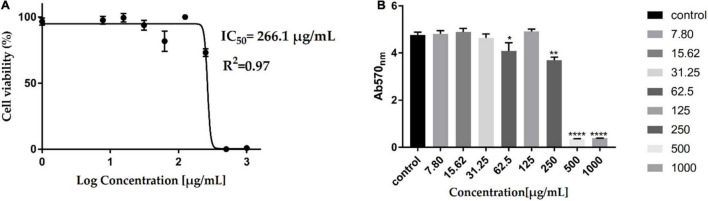
Cytotoxicity of AWME3 fat on HEK-293 cell lines. Cells were treated with serial of AWME3 dilutions for 24 h. Then, viability of AWME3 treated-cells was measured using MTT assay **(A,B)**. Where, **(A)** cell lines were treated with AWME3 for 24 h (X-axis: log concentrations of AWME3 extract from 0 to 1,000 (μg/mL) and Y-axis: the percentage of normalized absorbance). **(B)** The of average OD_570_ of the HEK-293 cell line treated with AWME3 for 24 h (Y-axis: the normalized absorbance of HEK-293 at OD_570_), X-axis: AWME3 concentrations from 0 to 1,000 μg/mL. The IC_50_ values were calculated using the non-linear regression mode of Graph pad Prism7 (Graph Pad Software Inc., San Diego, CA, United States). All Results are the mean (±SEM) from three independent experiments performed in triplicates (*n* = 8). Statistical values are indicated.

Assessment of the AWME3 sequentially extracted from *H. illucens* larvae fat for potential cytotoxicity (Figures 6A, B) is considered as an important step to evaluate its properties for further applications. The cytotoxic effect of AWME3 at 7.813–1,000 μg/ml concentration was investigated using HEK293 cells by MTT cell viability assay, which relies on mitochondrial metabolic capacity of viable cells. Cell viability results were obtained after 24 h of incubation with AWME3. It is obvious that cell viability decreased with increased concentration of the extract. AWME3 was toxic at a concentration higher than 500 μg/ml. However, cells exposed to lower extract concentrations (250 μg/ml) retained higher than 70% cell viability. The cytotoxic activity of AWME3 is capable of killing human pathogenic bacterial strains at low concentrations but did not kill eukaryotic HEK293 cells.

### Acidic Water Methanol Extract 3 Fatty Acid Profile

**FIGURE 7:**
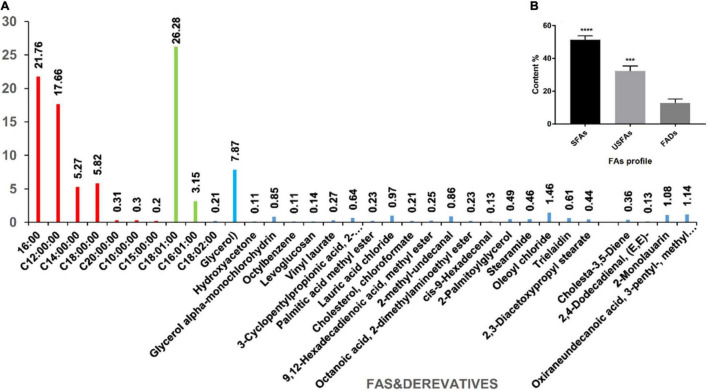
The composition of AWME3 extracted from *H. illucens* larvae fat under optimal conditions. **(A)** The percentages and the identity of the AWME3 chemical compounds detected by GC-MS analysis using the NIST-08 library; **(B)** The FAs profile that includes SFAs, USFAs, and FAs derivatives (FADs) statistically analyzed (***p = 0.0002, ****p < 0.0001) by one-way ANOVA.

This study evaluated the chemical composition of a new AWME3 batch obtained from the *H. illucens* larvae using our previously developed extraction and GC-MS analysis methods (Mohamed et al., 2021). The free fatty acids (FFAs) and their derivatives represented significant substances among 33 compounds identified by the GC-MS analysis of AWME3 (Supplementary Table 3 and Figure 7).

The major antibacterial components found in AWME3 were free fatty acids divided into saturated fatty acids (SFAs) and unsaturated fatty acids (USFAs). SFAs include palmitic (C16:0, 21.76%), lauric (C12:0, 17.66%), stearic (C18:0, 5.82%), myristic (C14:0, 5.27%), arachidic (C20:0, 0.31%), capric (C10:0, 0.3%), and pentadecyclic (C15:0, 0.2%) acids. USFAs contain three major free USFAs; moreover, cis-oleic acid (C18:1, 26.28%) is the most abundant among all the FA profiles followed by palmitoleic (C16:1, 3.15%) and linoleic acid (C18:2, 0.21%), as shown in Supplementary Table 3 and Figure 7.

The GC-MS analysis shows that the AWME3 extract is rich in molecules containing a hydrocarbon (hydrophobic) in the side chain in addition to cis or trans double bonds and polar functional groups (OH, NH2) that are hydrophilic. The AWME3 constituents contain OH^–^ and Cl^–^ ions believed to increase antibacterial activity. These ions are distributed in FA derivatives such as glycerol that was found in high concentration (7.87%); oxiraneundecanoic acid, 3- pentyl-, methyl ester, cis- (1.14%), lauric acid beta-monoglycerol (1.08%), 9,12-hexadecadienoic acid, methyl ester (0.25%), cis-9-hexadecenal (0.13%); and 2,4-dodecadienal, (E, E)- (0.13%).

## Discussion

The fast-growing number of MDR bacteria has increased and caused a big problem in treating bacterial diseases. Gram-negative bacilli, especially *K.pneumoniae*, are major problematic organisms that can resist different categories of antibiotics (Algammal et al., 2020d). *K. pneumoniae* has experienced increased resistance to various antibiotics, particularly strains with carbapenem enzymes that are highly virulent and acquire resistance to multiple antibiotics leading to high mortality rate in immunocompromised patients (Le et al., 2021). The *K. pneumoniae* strains KPM9 and KPi1627 have genes on both chromosomes and plasmid associated with virulence and antibacterial resistance identified in their genomes. Furthermore, these genes are responsible for the hypermucoviscosity of these strains. *K. pneumoniae* KPi1627 and *K. pneumoniae* KPM9 were reported to have K1 and K20 capsids responsible for virulence and pathogenicity (Lev et al., 2018). MDR can be conferred in bacteria *via* genetic mutation and horizontal gene transfer through chromosomes, plasmids, transposons, and other mobile genetic elements (Giedraitiene et al., 2011). In this study, a high rate of quinolone (levofloxacin and ciprofloxacin) resistance was evident in *K. pneumoniae* (33.3%), where *K. pneumoniae* ATCC BAA-2473 had MICs equal to 156 μg/ml, while the MBC values caused by levofloxacin and ciprofloxacin were 625 and 312.5 μg/ml, respectively. The resistance to fluoroquinolone groups among *K. pneumoniae* strains is mostly caused by mutations targeting quinolone resistance-determining regions (QRDR) of *gyr*A and *par*C of DNA gyrase and topoisomerase (Kareem et al., 2021). Thus, there is an immediate need for novel treatment modalities targeting MDR microbes (Bhatia et al., 2021).

Several studies on new antimicrobial agents have led to FAs being considered an alternative drug to combat MDR bacteria. The antibacterial properties of FAs have been known for a long time. Plants, algae, and animals produce FAs to defend against pathogens such as multidrug-resistant bacteria (Kabara et al., 1972; Venkata Mohan et al., 2015). Our previous studies have demonstrated that FAs extracted sequentially from *H. illucens* larvae have significant antimicrobial activity against MDR *Aeromonas* spp. and phytopathogenic bacterial strains (Marusich et al., 2020; Mohamed et al., 2021). In this study, we focused on the antimicrobial activity of AWME3 against severe MDR human pathogens, specifically clinically and environmentally isolated wild-type *K. pneumoniae* strains. The antibacterial properties of AWME3 were determined by measuring the IZD, MIC, MBC, and MIC50. The IZD caused by AWME3 against the *K. pneumoniae* KPi1627 and *K. pneumoniae* ATCC BAA-2473 strains were greater than those caused by the oil of *H. illucens* (9.6 ± 2.75 mm) against *S. aureus* determined by others (Saviane et al., 2021). A methanolic extract of *H. illucens* larvae caused IZD in the range of 7–9 mm against *K. pneumoniae* at 20 mg/ml. In addition, MIC50 values against *K. pneumoniae* strains were 22.37 and 39.28 mg/ml at 12 and 24 h, respectively (Saviane et al., 2021). Recent studies documented that linoleic acid (C18:2) inhibits the biofilm formation by *K. pneumoniae* ATCC 13883 (Hobby et al., 2019). In addition, linoleic acid increased the activity of polymixin B through the synergetic effect against *Pseudomonas aureginosa* (Baker et al., 2018). Linoleic and palmitoleic acids proved to block and inhibit enzyme streptokinase-mediated plasminogen and reduce the severity of invasive pathogenic *Streptococcus* group A (Rox et al., 2017). The resistance of *K. pneumoniae* ATCC BAA-2473 caused by several antibiotics during the second 12 h of incubation time in this study developed because of the expression of extended-spectrum beta-lactamases (ESBLs) or New Delhi Metallo-beta-lactamase 1 (NDM) (Hu et al., 2019). This study demonstrated that MIC50 values ranging between 126 ± 0.045–149.5 ± 0.013 μg/ml and 155.6 ± 0.009–157.1 ± 0.009 μg/ml at 12 and 24 h against both *K. pneumoniae* ATCC BAA-2473 and *K. pneumoniae* KPi1627, respectively. Thus, our results indicated that AWME3 is more potent than antimicrobial peptides or the methanolic extract of *H. illucens* larvae.

Although some of the new classes of antibiotics can target Gram-positive bacteria, the major challenge is still finding new antibiotics against Gram-negative bacteria, which are identified as a critical priority by the World Health Organization [WHO] (2017). Because of the complexity of the Gram-negative cell wall, discovery of novel antibiotics that can permeate this barrier and stay inside a bacterium is a big challenge (Silver, 2016). As resistance rates continue to rise, novel approaches for antimicrobial therapy for MDR bacteria, including carbapenem-resistant enterobacteria, will become increasingly critical. Some antibiotics against these pathogens have significant clinical drawbacks such as toxic side effects of colistin and poor distribution to specific important body sites, including blood and urine by tigecycline. Even novel agents, such as ceftazidime-avibactam, are vulnerable to the emergence of resistance (Karaiskos et al., 2019). Aminoglycosides like streptomycin and gentamycin are often used in combination with penicillin to treat severe nosocomial infections. Similar to aminoglycosides, amphenicols like chloramphenicol inhibit protein synthesis (Jain et al., 2021). In this study, we showed that extended exposure to AWME3 did not generate resistance of the *K. pneumoniae* strains, including standard NDM-1 carbapenemase-producing the ATCC BAA-2473 strain, along with the wild-type hypermucoviscous nosocomial isolate, strain *K. pneumoniae* KPi1627 and the environmental isolate, strain *K. pneumoniae* KPM9. All the tested strains were resistant to multiple antibiotics (MDR), as confirmed by our susceptibility assay (Table 1, Supplementary Figure 1, and Supplementary Tables 1, 2).

It is well-known that insect species, the rearing substrate, and the extraction method have a great influence on the quantity and quality of insect oil yield (Scala et al., 2020). This study used the fat isolated from larvae grown on wheat as the rearing substrate. Constituents of sequentially extracted AWME3 were analyzed by GC-MS (Supplementary Table 3 and Figure 7). Our results demonstrated that AWME3 has 33 different chemical compounds, of which the most commonly found are cis-oleic (26.28%), palmitic (17.66%), stearic (5.82%), myristic (5.27%), and palmitoleic (3.15%) acids. Our extraction strategy leads to the enrichment of the content and percentages of SFAs and USFAs in AWME3 by sequential extraction compared to the recent research by Hender et al. (2021). They determined that the FA profile in *H. illucens* larvae consists of palmitic (C16:0, 20.47%), myristic (8.87%), stearic (C18:0, 4.65%), cis-oleic (C18:1n-9, 23.37%), palmetoleic (C16:1n-7, 6.21%), and linoleic (C18:2, 0.21%) acids. The difference in the content and percentages of FA profile depends mainly on the substrate used to feed the larvae (Barragan-Fonseca et al., 2017). Fatty acids of HI larvae feed on palm kernel meal, and HI larvae reared on 80% industrial waste mix with 20% organic waste were almost the same. The most dominant FAs were lauric (40.54–46.72%), oleic (17.48–15.98%), palmitic (14.55–12.12%), and myristic (15.57–11.13%), for both HI larvae oils (Alifian et al., 2019). Accumulation of FAs and vitamins was reported (Liland et al., 2017), where omega-3 fatty acid eicosapentaenoic acid (20:5n-3), iodine, and vitamin E concentrations increased in the larvae when more seaweeds was included in the diet. Our findings followed the results obtained by Mai et al. (2019), who investigated and analyzed the FA composition of 7-day old BSF larvae fed with broiler and cow manure. Main fat constituents were capric (10:0, 0.8%), lauric (12:0, 31.9%), myristic (14:0, 5.5%), palmitic (16:0, 21.9%), stearic (18:0, 2.7%), palmitoliec (16:1, 2.3%), oleic (18:1, 20.2%), linoliec (18:2, 13%), and linolenic (18:3, 1.7%) acids. Furthermore, AWME3 became more enriched with FAs during successive extraction to give high percentages of SFAs and USFAs compared to the findings of Barragan-Fonseca et al. (2017), who detected FAs with content (10:0, 1.8%), (12:0, 23.4%), (14:0, 3.9%), (16:0, 10.5%), (18:0, 1.8%), (16:1, 2.9%), (18:1, 10.2%), (18:2, 3.22%), and linolenic acid (18:3, 3.6%).

It is known that the antimicrobial potential of fatty acids depends on hydrocarbon chain length, unsaturation, and presence of functional groups (Yoon et al., 2018). It was reported that Gram-positive bacteria are sensitive to fatty acids, and that very few Gram-negative species are sensitive to these molecules. We demonstrated that AWME3 was highly active against both Gram-positive bacteria (Marusich et al., 2020; Mohamed et al., 2021) and the MDR Gram-negative *K. pneumoniae* strains tested in this study. Gram-positive bacteria have a cell wall constituted by a single thick peptidoglycan layer only, while Gram-negative bacteria, in addition to a thin peptidoglycan layer, have an outer membrane layer composed of lipoproteins, lipopolysaccharides, and phospholipids (Godlewska et al., 2009). This layer of Gram-negative bacteria prevents the entry of intermediate and long-chain fatty acids and their subsequent toxic action (McGaw et al., 2002).

Nevertheless, our results were commensurate with the findings of fatty acid methyl ester extracts of the blind eye mangrove and lipophilic extracts of various parts of the plant *Pistacia vera* (Özçelik et al., 2005; Agoramoorthy et al., 2007). Some microalgae, such as *Chlorella vulgaris*, *Planktochlorella nurekis*, and *Ulva rigida*, produce chlorelin, a mixture of FAs that inhibit clinical and non-clinical MDR bacteria (Čermák et al., 2015; Shannon and Abu-Ghannam, 2016; Ismail et al., 2018; Potocki et al., 2021). El Shafay et al. (2016) isolated and identified antibacterial FAs from *Sargassum vulgare* and *Sargassum fusiforme* extracts, which killed MDR clinical isolates *S. aureus* and *K. pneumoniae*. The hexane extract of *Halimeda discoidea*, which contains FAs such as palmitic acid, inhibits *Klebsiella pneumoniae* ATCC 13883 (Supardy et al., 2012). These results were in line with ours.

The oleic, linoleic, and linolenic acids have the most potent antibacterial activity among long-chain unsaturated fatty acids (USFAs). In contrast, long-chain saturated fatty acids (SFAs), including palmitic and stearic acid, are less active (Zheng et al., 2005). *H. illucens* larvae fat contains a significant quantity of USFAs, especially cis-oleic acid (Supplementary Table 3 and Figure 7). In addition, *H. illucens* oil is characterized by a prevalent amount of lauric acid (Figure 7), a medium-chain fatty acid, and it has a broad spectrum of activity against different bacteria *in vitro* (Bergsson et al., 2001; Fischer et al., 2012). Matsue et al. (2019) demonstrated that lauric acid itself did not inhibit the *K. pneumoniae* ATCC 4352 strain even at 2.5 Mm. Thus, a combination of FAs enhances the activity of AWME3 to kill MDR *K. pneumonie* strains at 250 μg/ml. Skalicka-Woźniak et al. (2010) extracted FAs from fruits of *Peucedanum cervaria* and *P. alsaticum* that were less active against *K. pneumoniae* ATCC 13883, with MIC 0.5–4 mg/ml, compared to AWME3 MIC 250 μg/ml. The quantity and quality of fatty acids, especially in *H. illucens* larvae, can differ with progression of the larval instars and use of different rearing substrates (McGaw et al., 2002). However, BSFL oil always contains a good quantity of lauric acid. Poly USFAs and free fatty acids have also been identified as antimicrobials with a broad spectrum of activity not affected by classic resistance mechanisms (Desbois and Smith, 2010; Desbois, 2012; Desbois and Lawlor, 2013). The permeability of bacterial cell membranes is influenced by exogenous fatty acids, particularly those containing cis isomers. Incorporating cis UFAs such as linoleic acid (18:2) can disturb membrane dynamics, increasing membrane permeability. Furthermore, the first double bond location in USFAs may significantly affect membrane permeability (Hobby et al., 2019). Here, we demonstrated that the AWME3 FAs dose-dependently increased the relative conductivity of treated planktonic bacteria, suggesting leakage of bacterial electrolytes due to disruption of cell permeability caused by AWME3 treatment with the incubation time. Besides, Uppu et al. (2017) has stated that membrane-active agents against *K. pneumoniae* and different Gram-negative bacteria strains acted by disruption of bacterial membranes as a prime target for therapeutic attack. Thus, the fatty acid-induced alterations in *K. pneumoniae* membrane permeability observed in this study were confirmed by two permeability assays, consistent with our study.

Reports of AMPs extracted from *H. illucens* larvae being safe for normal human cell lines (Tian et al., 2018), as well as the fact that MICs of *H. illucens* larvae extract against *E. coli* and *S. aureus* were lower than those in normal animal cells were in line with our results (Lee et al., 2020). Four *Cameronian* plant extracts showed no toxicity when they were incubated for 48 h with HEK293 normal human kidney cell lines (Somaida et al., 2020). Lipids extracted from brains of silver carp have protective effects against oxidative damage in human HEK293 cells (Wang et al., 2017).

In summary, there is an urgent need for alternative antimicrobial agents for treatment of nosocomial infections caused by MDR *K. pneumoniae*. Bioactive sustainable compounds in *H. illucens* larvae have attracted attention as new alternative agents against these pathogens because of their potent antimicrobial action properties. Insect oils are rich in other putative antimicrobials such as antimicrobial peptides, polymers, and chemical complexes that could act alone or synergically with fatty acids (Tzompa-Sosa et al., 2014, 2019; Vogel et al., 2018; Nesa et al., 2019). We focused on the lipid part of *H. illucens* larvae, which might be an excellent promising valuable material for developing a new type of antibacterials. Our data suggest that the AWME3 sequentially extracted from the same biomass of *H. illucens* larvae fat has SFAs and USFAs containing cis-double bonds with bactericidal resistance-free effect and could be a beneficial therapeutic agent for the treatment of infectious diseases. Our new data on AWME3-mediated membrane permeability effect pave the way for ongoing studies on the mode of FA action in detail. Furthermore, *in vivo* AWME3 toxicity studies will embark and recommend the use of these natural compounds in humans or animals and many pharmaceutical and multiple industrial applications.

## Conclusion

In this study, we demonstrated for the first time that the sequentially extracted AWME3 from *H. illucens* larvae fat displays bactericidal activity against MDR Gram-negative *K. pneumoniae* strains. Notably, impairment of bacterial outer membrane permeability underlies the AWME3-mediated bactericidal effect that did not generate resistance while being non-toxic in human embryonic kidney cells. Together, our results suggest a promising effective therapeutic strategy based on AWME3 with minimal risk of antimicrobial resistance to combat antibiotic-tolerant severe infections, including nosocomial ones.

## Data Availability Statement

The original contributions presented in the study are included in the article/Supplementary Material, further inquiries can be directed to the corresponding authors.

## Author Contributions

HM designed and implemented all the experiments, analyzed all the data, wrote the original draft, and analyzed the GC-MS data. EM conceived, designed and supervised the study, and edited and revised the manuscript. HM and YA conducted all the GC-MS experiments. SL designed and supervised the study, edited and revised the manuscript, administrated the project, and procured the funding. All authors have read and agreed to the published version of the manuscript.

## Conflict of Interest

The authors declare that the research was conducted in the absence of any commercial or financial relationships that could be construed as a potential conflict of interest.

## Publisher’s Note

All claims expressed in this article are solely those of the authors and do not necessarily represent those of their affiliated organizations, or those of the publisher, the editors and the reviewers. Any product that may be evaluated in this article, or claim that may be made by its manufacturer, is not guaranteed or endorsed by the publisher.

## Abbreviations

NDM-1: New Delhi metallo-B-lactamase-1
KPi1627: *Klebsiella pneumoniae* subsp. *pneumoniae* i1627
KPM9: *Klebsiella pneumoniae* subsp. *pneumoniae* M9
KP ATCC BAA-2473: *Klebsiella pneumoniae* ATCC BAA-2473
DMEM: Dulbecco’s Modified Eagle Medium
MTT: 3-(4,5-dimethylthiazol-2-yl)-2,5-diphenyltetrazolium bromide
AWME3: acidic water methanol extract 3
FFAs: free fatty acids
SFAs: saturated fatty acids
USFAs: unsaturated fatty acids
REC: relative electric conductivity
HI: *Hermetia illucens*
BSFL: black soldier fly larvae
hvKP: hypervirulent *Klebsiella pneumoniae*
IZD: inhibition zone diameter
MDR: multidrug-resistant
MH: Muller-Hinton
LB: Lauria Bertani
MIC: minimum inhibitory concentration
MBC: minimum bactericidal concentration
MIC50: half of minimum inhibitory concentration
GC-MS: gas chromatography-mass spectrometry
ATCC: American-type culture collection.
