# Supplementary material for: Bacterial Outer Membrane Permeability Increase Underlies the Bactericidal Effect of Fatty Acids From Hermetia illucens (Black Soldier Fly) Larvae Fat Against Hypermucoviscous Isolates of Klebsiella pneumoniae
Source: Front Microbiol. 2022 May 6;13:844811. doi: 10.3389/fmicb.2022.844811 (PMC9121012; doi:10.3389/fmicb.2022.844811)
Supplement: Supplementary file 1 [file Data_Sheet_1.pdf]

Time	
**Time**	**(A)** ***K. pneumoniae*** **KPM9**
**12h**	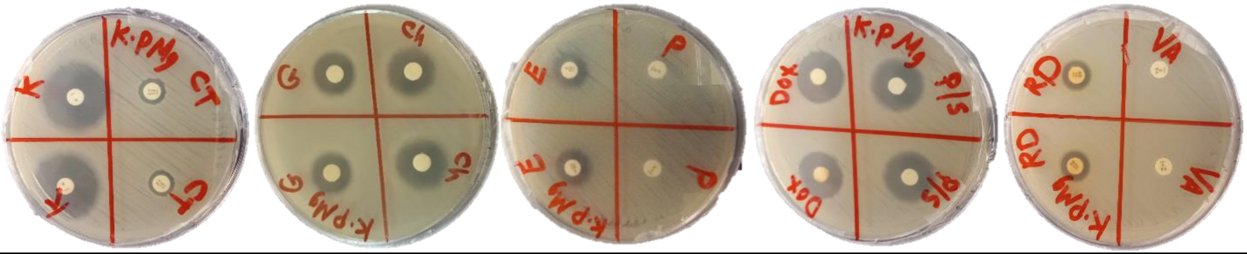
**24h**	**(B)** ***K. pneumoniae*** **KPM9**
	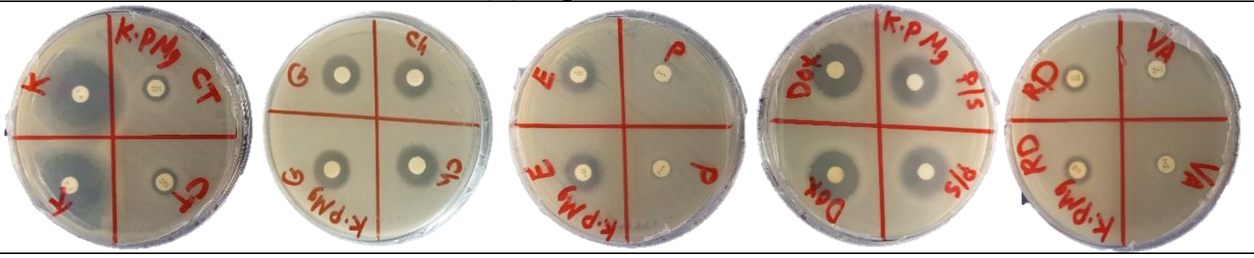
**12h**	**(C)** ***K. pnemoniae*** **KPi1627**
	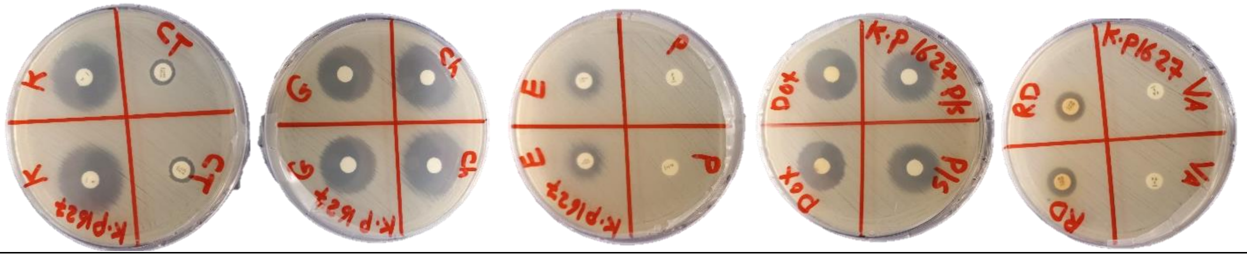
**24h**	**(D)** ***K. pnemoniae*** **KPi1627**
	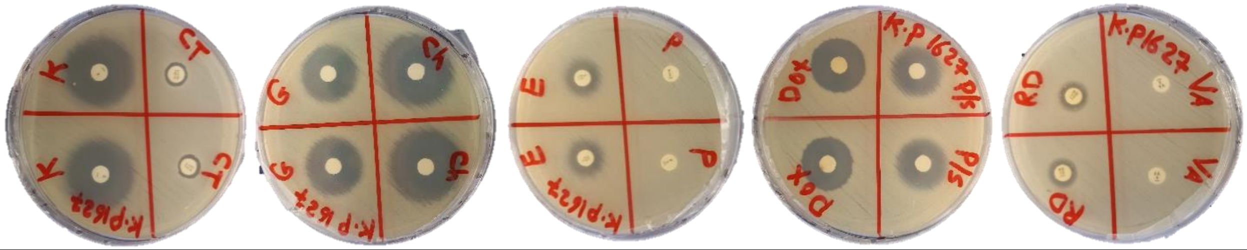
**12h**	**(E)** ***K. pnemoniae*** **ATCC BAA-2473**
	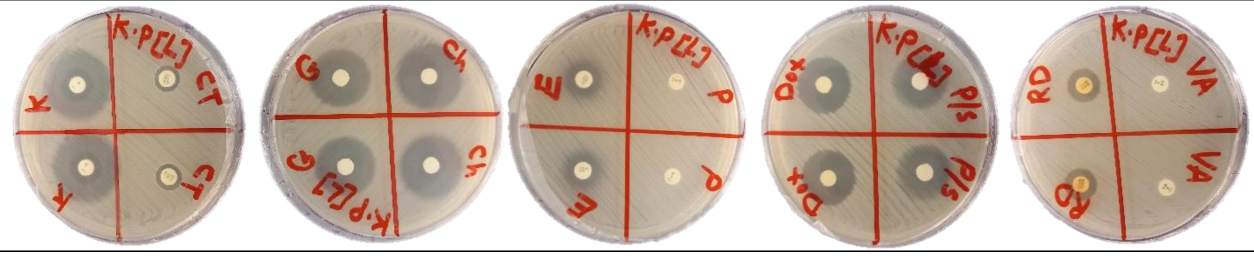
**24h**	**(F)** ***K. pnemoniae*** **ATCC BAA-2473**

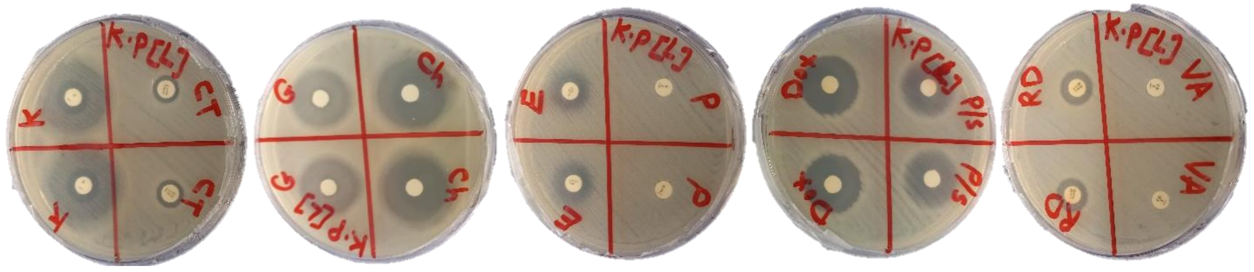

Supplementary Figure S1. Susceptibility of **(A, B)** *K. pneumoniae* KPM9, **(C, D)** *K. pnemoniae* KPi1627 and **(E, F)** *K. pnemoniae* ATCC BAA-2473 against ten groups of antibiotics determined by disk diffusion assay. All antibiotics were applied to the discs and gently pressed on the surface of the Muller Hinton agar Petri dishes with cultured bacteria strains at density 10^8 (CFU/mL). All discs were placed in duplicates, and the mean of resulting IZD ±SD of three independent experiments was estimated.

Supplementary Table S1. MICs, MBCs, and MBC/MIC ratio of different antibiotics against human pathogenic *K. pneumoniae* strains.

**Antibiotics**	***K. pneumoniae* KPi1627**			***K. pneumoniae* KPM9**			***K. pneumoniae* ATCC-2473**			**MIC breakpoints (µg/ml) recommended by CLSI**		
	MIC	MBC	MBC /MIC	MIC	MBC	MBC /MIC	MIC	MBC	MBC /MIC	S	I	R
G	2.25 (S)	2.25	1	1.56 (S)	3.12	2	≥1250 (R)	≥1250	-	≤4	8	≥16
Ch	6.25 (S)	25	4	6.25 (S)	50	8	39 (R)	156	4	≤8	16	≥32
K	12.5 (S)	25	2	12.5 (S)	12.5	1	≥1250 (R)	≥1250	-	≤16	32	64
DOX	1.56 (S)	6.25	4	3.12 (S)	12.5	4	6.25 (I)	50	8	≤4	8	≥16

P/S	9.77 (S)	9.77	1	9.77 (S)	9.77	1	≥1250 (R)	≥1250	-	-	-	-
CT	≥1250 (R)	≥1250	-	11.33 (R)	11.33	1	0.39 (S)	1.56	4	≤2	-	≥4
RD	78 (R)	312.5	4	39 (R)	78	2	≥1250 (R)	≥1250	-	≤1	2	≥4
E	625 (R)	625	1	625 (R)	1250	2	625 (R)	≥1250	-	≤0.5	1-4	≥8
P	312.5 (R)	312.5	1	625 (R)	625	1	≥1250	≥1250 (R)	-	≤0.12	-	≥0.25
Amp	156.25 (R)	625	4	156.25 (R)	312.5	2	≥1250 (R)	≥1250	-	≤8	16	≥32
Lev	0.39 (S)	0.39	1	0.098 (S)	0.195	2	156 (R)	625	4	≤2	4	≥8
Cip	0.39 (S)	0.39	1	0.39 (S)	0.39	1	156 (R)	312.5	2	≤1	2	≥4
Cef	1.56 (S)	6.25	4	0.08 (S)	0.08	1	25 (R)	100	4	≤2	-	≥16

-Values obtained were compared with those recommended by the CLSI

-Abbreviations: G, gentamicin; Ch, chloramphenicol; K, kanamycin; DOX, doxycycline; P/S, penicillin-streptomycin; CT, colistin; RD, rifampicin; E, erythromycin; VA, vancomycin; P, penicillin; Amp, Ampicillin; Lev, levofloxacin; Cip, ciprofloxacin; Cef, cefepime; R, resistant; I, intermediate; S, susceptible; (0), means no inhibition zone around the disc on the plate; (-), not determined.

Supplementary Table S2. Antimicrobial resistance pattern of human pathogenic *K. pneumoniae* strains ($n = 3$).

**Antibiotic classes**	**Specific antibiotic**	**Interpretation**					
		Sensitive		Intermediate		Resistance	
		N	*%*	*N*	*%*	*N*	*%*
Aminoglycosides	Gentamycin	2	66.7	-	-	1	33.3
	Kanamycin	2	66.7	-	-	1	33.3
	Penicillin-streptomycin	2	66.7	-	-	1	33.3
Ansamycins	Rifampicin	-	-	-	-	3	100
Glycopeptides	Vancomycin	-	-	-	-	3	100
Macrolides	Erythromycin	-	-	-	-	3	100
Tetracyclines	Doxycycline	2	66.7	1	33.3	-	-
Fluroquinolones	Ciprofloxacin	2	66.7	-	-	1	33.3
	Levofloxacin	2	66.7	-	-	1	33.3
Phenicols	Chloramphenicol	2	66.7	-	-	1	33.3
Penicillins	Penicillin	-	-	-	-	3	100
	Ampicillin	-	-	-	-	3	100
Lipopeptides	Colistin	1	33.3			2	66.7
Cephems	Cefepime	2	66.7	-	-	1	33.3
P value		$P < 0.0001$		$P < 0.0001$		$P < 0.0001$	

<table>
<tr><th>Time</th><th>(a) K. pneumoniae KPi1627</th></tr>
<tr><td>12 h</td><td>(A) (B) (C)
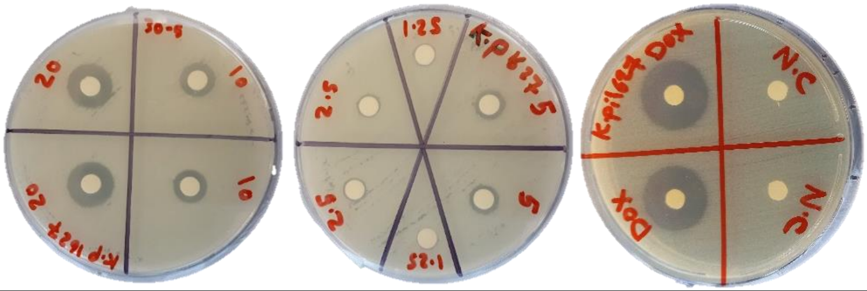
</td></tr>
<tr><td>24</td><td>(b) K. pneumoniae KPi1627</td></tr>
<tr><td></td><td>(A) (B) (C)
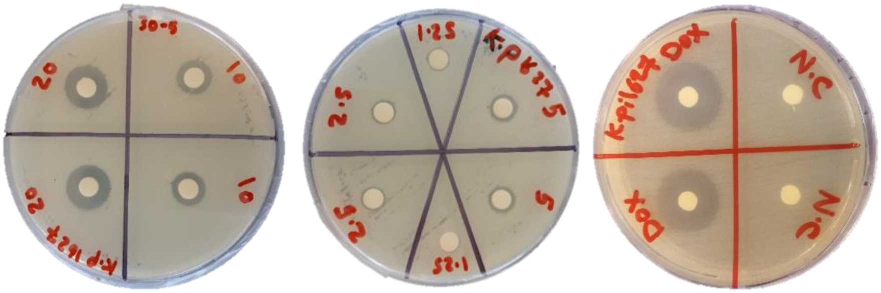
</td></tr>
<tr><td>12h</td><td>(c) K. pneumoniae KPM9</td></tr>
<tr><td></td><td>(A) (B) (C)
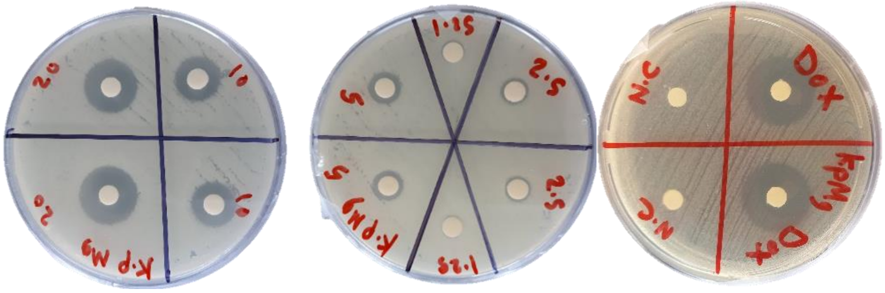
</td></tr>
<tr><td>24h</td><td>(d) K. pneumoniae KP M9</td></tr>
<tr><td></td><td>(A) (B) (C)</td></tr>
</table>

	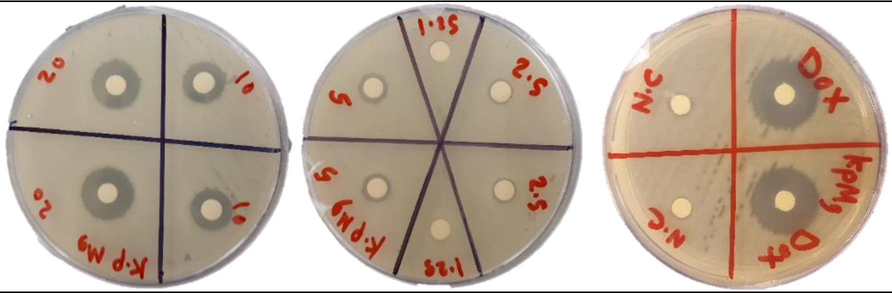
**12h**	**(e)** *K. pneumoniae* **ATCC BAA-2473**
	**(A)** **(B)** **(C)** 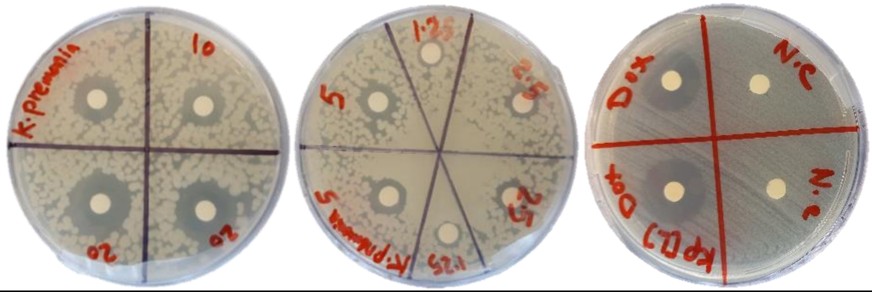
**24h**	**(f)** *K. pneumoniae* **ATCC BAA-2473**
	**(A)** **(B)** **(C)** 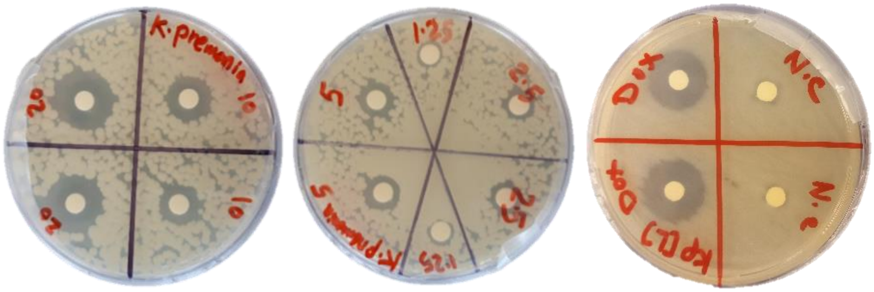

Supplementary Figure S2. The inhibition zone assay for tested bacteria strains. The inhibition zone diameters were measured after 12 h and 24 h of overnight incubation of the discs loaded on Petri dishes agar seeded with (a, b) *K. pneumoniae* KPi1627; (c, d) *K. pneumoniae* KPM9*;* and (e, f) *K. pneumoniae* ATCC BAA-2473 strains with suspension 5× 10^8 CFU/mL; **(A)** The discs were loaded with 50 µL of AWME3 from larvae fat with concentrations 10 and 20 mg/mL; **(B)** 50 µL of AWME3 loaded with 1.25, 2.5 and 5 mg/mL; **(C)** 50 µL of DOX with concentration 600 µg/mL (30 µg/disk) was used as positive control and 50 µL of pure AWM extracting reagent was used as a negative control (N.C). All samples were loaded in duplicates.

Supplementary Table S3. GC-MS analysis and identification of the major compounds in AWME3 of *H. illucens* larvae fat.

Common name		C: D	CAS	Content %	RT	Structure
**SFAs**	Palmitic acid	16:0	57-10-3	21.76	15.286	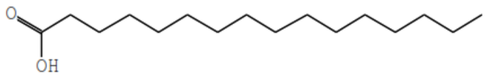
	Lauric acid	12:0	143-07-7	17.66	12.331	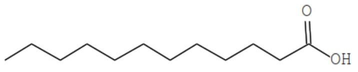
	Myristic acid	14:0	544-63-8	5.27	14.058	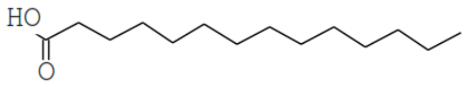
	Stearic acid	18:0	57-11-4	5.82	16.439	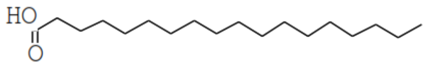
	Arachidic acid	20:0	506-30-9	0.31	17.345	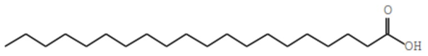
	Capric acid	10:0	334-48-5	0.3	10.403	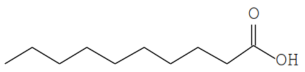
	Pentadecylic acid	15:0	1002-84-2	0.2	14.753	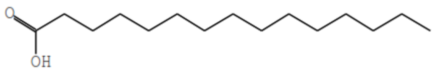
**USFAs**	cis-Oleic acid	18:1	112-80-1	26.28	16.35	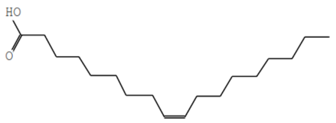
	Palmitoleic acid	16:1	2091-29-4	3.15	15.286	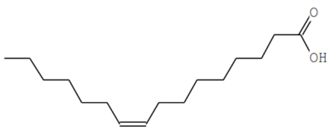

	Linoleic acid	18:2	60-33-3	0.21	16.723	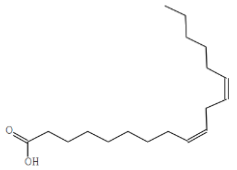
**FAs Derivatives**	Glycerol)	-	56-81-5	7.87	6.808	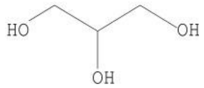
	Hydroxyacetone	-	116-09-6	0.11	3.798	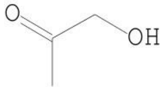
	Glycerol alpha-monochlorohydrin	-	96-24-2	0.85	5.928	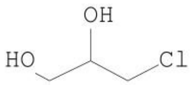
	Octylbenzene	-	2189-60-8	0.11	11.552	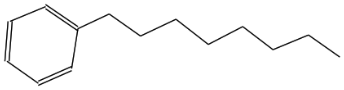
	Levoglucosan	-	498-07-7	0.14	11.84	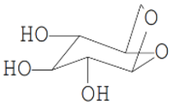
	Vinyl laurate	-	146-71-6	0.27	14.683	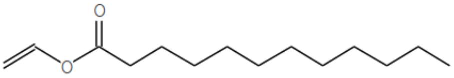
	3-Cyclopentylpropionic acid, 2-dimethylaminoethyl ester	-	0-00-0	0.64	14.936	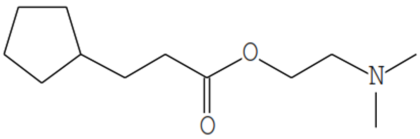
	Palmitic acid methyl ester	-	112-39-0	0.23	15.187	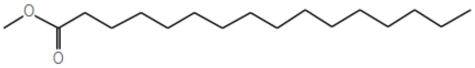
	Lauric acid chloride	-	112-16-3	0.97	15.55	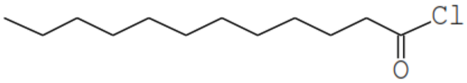

	Cholesterol, chloroformate	-	7144-08-3	0.21	15.761	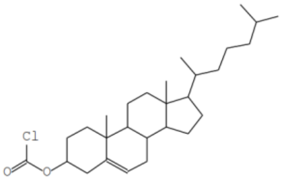
	9,12-Hexadecadienoic acid, methyl ester	-	2462-80-8	0.25	16.542	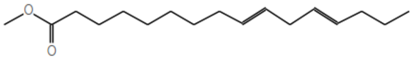
	2-methyl-undecanal	-	110-41-8	0.86	17.004	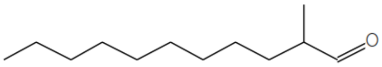
	Octanoic acid, 2-dimethylaminoethyl ester	-	0-00-0	0.23	17.82	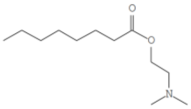
	cis-9-Hexadecenal	-	56219-04-6	0.13	17.901	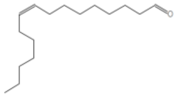
	2-Palmitoylglycerol	-	23470-00-0	0.49	18.101	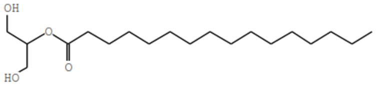
	Stearamide	-	124-26-5	0.46	18.55	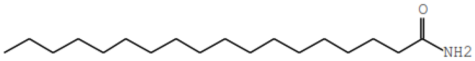
	Oleoyl chloride	-	112-77-6	1.46	19.01	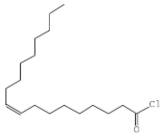
	Trielaidin	-	537-39-3	0.61	19.163	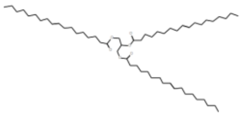
	2,3-Diacetoxypropyl stearate	-	56846-96-9	0.44	20.3	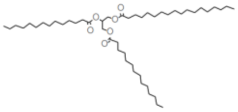
	Cholesta-3,5-Diene	-	747-90-0	0.36	21.136	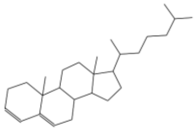

	2,4-Dodecadienal, (E,E)-	-	56219-04-6	0.13	13.923	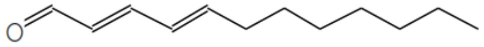
	2-Monolauarin	-	1678-45-1	1.08	16.021	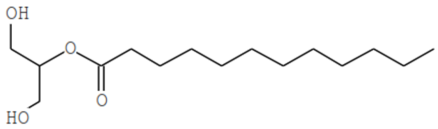
	Oxiraneundecanoic acid, 3-pentyl-, methyl ester, cis-	-	38520-30-8	1.14	16.137	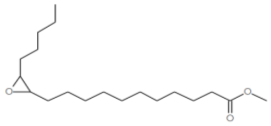

CAS: Chemical abstract service RT: Retention time C: D, where C is the number of carbon atoms and D is the number of double bonds in the carbon chain.
